# From a dream to a resounding reality: the inception of a doctors union in Kenya

**Published:** 2012-01-25

**Authors:** Aruyaru Stanley Mwenda

**Affiliations:** 1 Consolata Hospital, Nyeri, Kenya

**Keywords:** Doctor, union, strike, health workforce, Kenya

## Abstract

After two grueling years of determination and resilience, the Kenyan doctors have formed a doctors’ union. The Kenya Medical Practitioners, Pharmacists and Dentists Union(KMPDU)not only aims at agitating for better terms of service for its members, it also aims to influence the running of the Kenyan health sector and improve health services in Kenya. The union has been fully recognized by the Kenyan government and is engaging the government concerning various health sector policies besides improved terms and working conditions of the doctors in the country.

## Introduction

Kenya has two established public medical schools (Moi University and University of Nairobi) and a private medical school (Aga Khan University). Kenyatta, Egerton and Maseno universities are new entrants and their graduates will soon be joining the Kenyan health sector. These universities churn out doctors every year into the labour (health) market. Even with these Kenya still has an abject shortage of doctors with a total of short of 8000 doctors, of which only 2300 are in the public hospitals to cater for majority of her population of approximately 40million [1]. This leaves Kenya in a desperate situation as far as meeting the world health organisation (WHO) recommendation of a doctor to patient ratio of 1:1000 [2] is concerned. Recent statistics have left the country far from achieving the health related millennium development goals [3]. Majority of Kenyan trained doctors have had to leave the public hospitals to join either the private health sector or travel abroad to seek a better life. The government of Kenya, in the wake of a recent doctors’ strike, has acknowledged that up to three quarters of doctors will have left the government payroll three years after joining the public health sector [4].

There are a myriad of bedevilments to the health sector in Kenya that have precipitated this. In the past, it has depended on the Kenyan health technocrats and political good will to appropriately address problems affecting Kenyans in the field of health. Despite Kenya being a signatory to the 2001 Abuja declaration [5] the treasury allocated way less than 15% of the national budget to healthcare in the 2011/2012 financial year [6].

There has been no union in the country that could stand up to the government and agitate for better funding of the healthcare. Doctors’ grievances have just existed with no formal body to address them.

In this article, the author-a member of the Kenya Medical Practitioners, Pharmacists and Dentists Union (KMPDU) who has keenly followed the developments during the entire period of formation of the union- highlights the cardinal issues that necessitated the formation of the union, the steps towards a functional union and the various challenges that were encountered during the entire process. This article is not intended as an analysis of the Kenyan health care but may just mention a few aspects that are relevant to this topic.

## Precipitants to formation of the doctors’ union

As noted above, there are several bedevilments that face the Kenyan healthcare system, especially the public sector healthcare. These are captured in a list of concerns and demands sent to the Kenyan ministries of medical services and public health and sanitation by KMPDU [7]. This article does not discuss the issues as raised but just highlights them.

### Shortage of health facilities

In Kenya, patients often have to walk long distances to access specialised treatment. Radiotherapy for various types of cancer for instance is only accessible in the capital city while dialysis, a basic requirement for patients in renal failure, is available in selected few centres in the country, way out of sync with the high number of patients requiring such intervention.

### Inadequate medical equipment

The doctors have decried the gross inadequacy of basic essentials in the various hospitals in Kenya. They have felt that they continued to watch as patients died because of lack of basic equipments and other medical aids on top of scarce drugs [8].

### Poor staffing

Kenya is way below the recommended WHO doctor to patient ratio of 1 in 1000 [2]. With approximately 40 million Kenyans, Kenya requires 40,000 doctors to meet that ratio. However the current total number of doctors is approximately 8,000 with only approximately 2300 of them being in the public health sector. Even among the few in the public health sector, majority tend to conglomerate in the urban centres leaving rural Kenya in a perpetual health crisis [9].

### Staff training

Two years ago, the government withdrew its sponsorship for the postgraduate training of doctors. This meant that in the setting of a serious shortage of specialists in the country, Kenya had to wait for doctors to sponsor themselves through post graduate training and somehow hope to fill the glaring deficits. This decision has however been receded in the wake of a recent doctors’ strike in the country. Its likely such a scenario would have dragged on into eternity were it not for the KMPDU which continuously agitated for the recession of that decision and even rallied all public health doctors to a country wide strike.

### Inhuman working conditions of residents in the Kenyan teaching hospitals

In the two teaching hospitals in Kenya ( Kenyatta National Hospital and Moi Teaching and Referral Hospital), residents who have sponsored themselves or are sponsored by non-governmental organisations (NGOs) work for long hours, day and night when on call, for no pay. This has been interpreted by the Kenyan doctors to be nothing but slavery. This continues to happen even as the concerned hospitals continue to generate income from the services of these specialists in training who literally run these hospitals on a day to day basis, sometimes even remaining behind when universities have closed.

### Unsatisfactory performance by ministry staff

There have been anecdotal concerns by the Kenyan doctors that the ministry of public health and sanitation and that of medical services are staffed with administrative individuals who are bent on punishing the Kenyan doctor as opposed to motivating them to work under the difficult circumstances. The Kenyan doctors perceive the poor policies and the suboptimal implementation of already existing health related policies (such as the budgetary allocation to health in accordance with the Abuja declaration) can be attributed to poor performance by these staff.

### Poor remuneration of doctors and unfair working conditions

Today, Kenya remains among the able Eastern African economies where doctors are poorly remunerated. They for example lack a special health scheme other than the national hospital insurance whose coverage the doctors decry is insufficient. This is in spite of the continuous danger of occupational injuries that they face daily. A new graduate medic in Kenya enters the public service with a basic salary of 30,000 Kenyan Shillings. Hardship allowance for the doctors working along the risky Kenya-Somalia, Kenya-Sudan borders is a mere 600 Kenyan Shillings. All these have led to the continual migration of Kenyan doctors to South Africa, Botswana and Namibia where remuneration is better. Chankova and colleagues found that resignations accounted for up to 80% of doctor attrition in the Kenyan public hospitals [9].

The government itself late 2011 admitted that up to three quarters of doctors joining public hospitals after graduation will have left these hospitals in three years’ time [4]. The Kenyan doctors thought they could not continue to leave poor Kenyans dying in public hospitals while they sought greener pastures outside the country, at least not without trying to correct the anomaly.

### Need for the formation of a health service commission

The poor pay among the Kenyan doctors is partly because of their lumping up with the rest of the public servants. Thus a doctor doing 72 hours of weekend shift in a busy provincial hospital is remunerated exactly the same way as a departmental secretary who works for eight hours a day, five days a week. Other unique cadres of workers in Kenya have service commissions that draw their budget, formulate terms of service and handle other welfare related issues. The judiciary staffs for example, are employed by the judicial service commission while the teachers are employed by the teachers’ service commission. The doctors of Kenya have thought that a health service commission would be best suited to conglomerate all the health sector staff and draw up their terms of service in a manner sensitive to the special requirements of this sector.

## Steps leading to the formation of the doctors’ union and the challenges encountered

The KMPDU began as a Facebook^TM^ group formed by young doctors and final year medical students in 2009. As was captured by the group's name “*United against the poor pay of doctors in Kenya*”, this group was initially focused on the poor pay especially among the young doctors who have not yet specialised and thus do not have the advantage of having part time consultancies where they would eke out an additional coin. These are the doctors who are the first on call in our hospitals, doing almost all the surgeries and other procedures, only preserving the complicated ones for the specialists. The idea of formation of a trade union for the doctors was not feasible at this point in time in view of the labour laws in Kenya which categorised doctors in Kenya as having managerial positions and thus being un-unionisable. The labour relations act 2007 also meant doctors could not picket, taking cognisance of their provision of essential services [10]. As such the plan by these young Kenyan doctors was to recruit as many young doctors as possible into Kenya Medical Association (KMA) and later use their majority in the association to call a special general meeting, hold new elections and eventually take control of the KMA leadership. This way, they would use the association to negotiate and pressure the employer to improve the terms of service for the doctors.

As they would later realise though, despite significant enrolment into the association, this was an entity that did not have a legal mandate for such a fight [11]. It was a glaring realization. There were two options left; forming a trade union or retaining the status quo. These young doctors chose the former. And so began a series of meetings. In October 2010, the doctors, mostly around Nairobi and central Kenya organised a meeting in Thika town. The agenda included a petition about the government's decision to stop funding the post graduate training. There were 60 doctors in that meeting. Resolutions were made, more meetings were scheduled, the media covered the event and one of the vocal doctors was interdicted. The way forward was to meet in a central park in Nairobi city (Uhuru Park) and do a peaceful protest. The turn up for this meeting was discouraging, but the doctors did not relent, they presented a petition to the Kenyan prime minister.

This was happening in the dawn of a new constitutional dispensation in Kenya which brought along a strong bill of rights, giving every Kenyan worker the freedom to join a trade union and compelling every employer to recognise employee's trade unions [12]. So after consultation with trade union experts, the doctors decided to form their own union. What followed were meetings, sometimes under the shelter of a petrol station on Sunday afternoons, to lay down the foundations of a trade union-all on volunteer basis. It was a mammoth task. Communication was through the same Facebook^TM^ group and all these doctors depended on was goodwill, hope and the few encouraging comments on the social media. None of them had seen a physical meeting of 61 doctors committed to their course! As the chairman of KMPDU would later observe in one of his many communiqués to the members, “...it is not an easy thing to unite doctors I can tell you”.

After a long run, the union was finally registered in august 2011. It embarked on massive final lap recruitment of more members and seeking of recognition from the government [6]. It is now fully recognised by the government of Kenya and continues to seek recognition from other employers in the country.

## What is the way forward for Kenyan healthcare in the dawn of the new union?

This union has galvanised the Kenyan doctors in a way no one had anticipated. It is no longer a union of the junior doctors. It is a union with membership across the age and specialty spectrum.

Although this is out of the context of this paper, the union is just from organising the most successful doctors’ strike in the history of Kenya. It is now in negotiations with the government; allowances for the doctors are being looked into, a bill to establish a Health Service Commission is in its drafting stage, discussions are advanced on the modalities of remunerating the doctors in post graduate training. Doctors are in constant meetings countrywide brainstorming on how to improve the various ills in the Kenyan health sector. The Kenyan doctors have woken up from their laid back position and are now determined, more than ever, to influence the running of the Kenyan health care system

## Conclusion

The formation of the doctors’ union in Kenya was a long tedious and highly involving undertaking. It took the determination of a set of doctors, withstanding all manner of criticism-some of it from their colleagues. Now that this union is a reality, it portends good not only for the Kenyan doctors but the Kenyan patient and, by extension, the entire Kenyan health care.
